# Traumatic Events and Health: An Ecological and Life Course Perspective

**DOI:** 10.1093/geroni/igab046.1650

**Published:** 2021-12-17

**Authors:** XinQi Dong

## Abstract

During the past decades, researchers have shown an increasing interest in the study of traumatic events among aging populations. The majority of studies on trauma focus on mental health, which overlooks the possibility that trauma may also have an adverse effect on other health outcomes, such as cognitive function. A number of studies focus on a single traumatic event. However, this approach may underestimate its health impact as many people experience multiple forms of traumatic events. Indeed, the impact of traumatic events on health depends on the event itself (e.g., single or multiple forms, time) as well as ecological factors. This symposium aims to address the above limitations. The first longitudinal study An Ecological Model of Risk Factors in Elder Mistreatment (EM) Victims tested different dimensions of the ecological model to prevent recurrence of EM. The second study Polyvictimization and Cognitive Function in an Ethnic Minority Aging Population explored whether exposure to multiple forms of EM affects cognitive function. The third study Traumatic Events and Cognitive Function: Does Time Matter? examined whether traumatic events happened in childhood, adulthood, or old age will influence late-life cognitive function. The fourth study Face-saving and Help-seeking among Older Adults with EM identified cultural determinants of help-seeking behaviors in EM victims. This symposium will advance knowledge in the health consequences of polyvictimization and exposure to traumatic events in different life stages. It will also inform interventions to stop the recurrence of EM in immigrant families and enhance the help-seeking behaviors of ethnic minority older adults.

